# Computational Modeling of Gluteus Medius Muscle Moment Arm in Caviomorph Rodents Reveals Ecomorphological Specializations

**DOI:** 10.3389/fbioe.2022.806314

**Published:** 2022-05-25

**Authors:** Lukas Löffler, Jan Wölfer, Flavia Gavrilei, John A. Nyakatura

**Affiliations:** AG Vergleichende Zoologie, Institut für Biologie, Humboldt-Universität zu Berlin, Berlin, Germany

**Keywords:** moment arms, locomotion, modeling, hind limb, Caviomorpha, hip joint

## Abstract

Vertebrate musculoskeletal locomotion is realized through lever-arm systems. The instantaneous muscle moment arm (IMMA), which is expected to be under selective pressure and thus of interest for ecomorphological studies, is a key aspect of these systems. The IMMA changes with joint motion. It’s length change is technically difficult to acquire and has not been compared in a larger phylogenetic ecomorphological framework, yet. Usually, proxies such as osteological in-levers are used instead. We used 18 species of the ecologically diverse clade of caviomorph rodents to test whether its diversity is reflected in the IMMA of the hip extensor M. gluteus medius. A large IMMA is beneficial for torque generation; a small IMMA facilitates fast joint excursion. We expected large IMMAs in scansorial species, small IMMAs in fossorial species, and somewhat intermediate IMMAs in cursorial species, depending on the relative importance of acceleration and joint angular velocity. We modeled the IMMA over the entire range of possible hip extensions and applied macroevolutionary model comparison to selected joint poses. We also obtained the osteological in-lever of the M. gluteus medius to compare it to the IMMA. At little hip extension, the IMMA was largest on average in scansorial species, while the other two lifestyles were similar. We interpret this as an emphasized need for increased hip joint torque when climbing on inclines, especially in a crouched posture. Cursorial species might benefit from fast joint excursion, but their similarity with the fossorial species is difficult to interpret and could hint at ecological similarities. At larger extension angles, cursorial species displayed the second-largest IMMAs after scansorial species. The larger IMMA optimum results in powerful hip extension which coincides with forward acceleration at late stance beneficial for climbing, jumping, and escaping predators. This might be less relevant for a fossorial lifestyle. The results of the in-lever only matched the IMMA results of larger hip extension angles, suggesting that the modeling of the IMMA provides more nuanced insights into adaptations of musculoskeletal lever-arm systems than this osteological proxy.

## 1 Introduction

**FIGURE 1:**
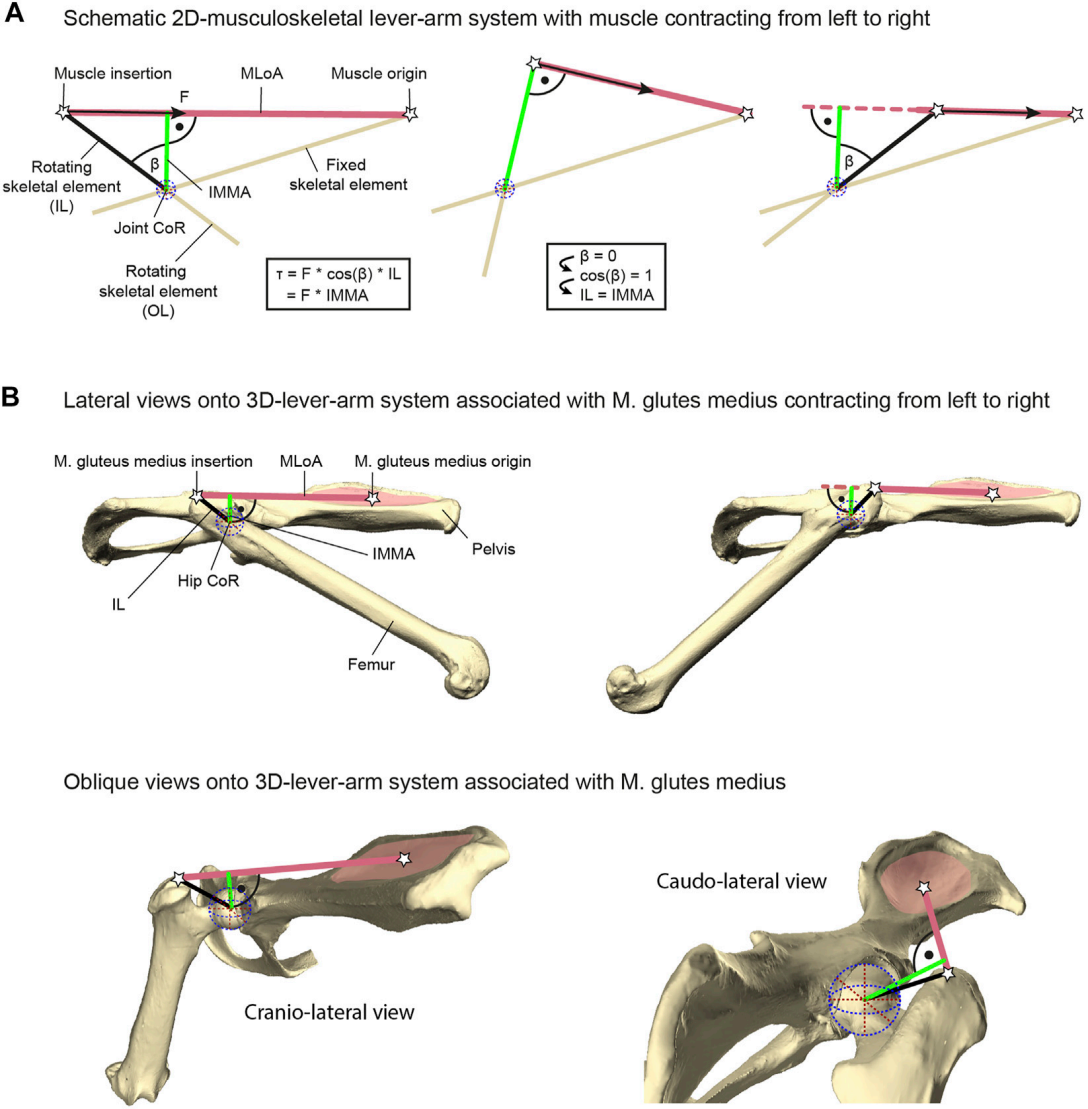
Explanation of lever-arm system components exemplified with the herein studied M. gluteus medius system. **(A)** Geometric properties of a schematic musculoskeletal lever-arm system as typically illustrated in a 2D plane, including the instantaneous muscle moment arm (IMMA), osteological in-lever (IL), osteological out-lever (OL), center of rotation (CoR), and the muscle’s line of action (MLoA). The skeletal element that includes the IL is modeled as being rotated due to muscle contraction with respect to a reference element fixed in space (left to right). The equation on the left shows different ways of computing the joint torque (τ) generated by the muscle’s force (F) and applies to all poses throughout joint motion. **(B)** 3D-lever-arm system associated with the M. gluteus medius exemplified using the surface models of the right femur and right half of the pelvis from the scans of *C. pilorides* (Desmarest’s hutia). Upper panels display lateral views onto the 3D model setup with a hip extended by 30° (left) and 140° (right) at 0° abduction (compare with left and right panels of **(A)**, respectively). Lower panels display oblique views onto the model setup with a hip extension of 120° at 0° abduction to highlight the actual 3D nature of the lever-arm system associated with the M. gluteus medius. For further explanation, see text.

The torque a muscle generates, for example, during vertebrate locomotion, is defined as the product of the muscle force acting along the muscle’s line of action (MLoA) and the length of the muscle moment arm (Pedotti et al., 1978; Murray et al., 2002; Pandy and Andriacchi, 2010). This moment arm is the perpendicular and, thus, the shortest distance from the considered MLoA to the joint’s center of rotation (CoR) of the joint the muscle is acting on (Figure 1). Muscle moment arms have been recognized to instantaneously change in accordance with joint movements due to the associated changes of the MLoA relative to the CoR (e.g., Lieber and Shoemaker, 1992, 1997). Hence, the functioning of this instantaneous muscle moment arm (IMMA) can only be understood by determining its length throughout relevant joint poses. For example, this can be achieved with the help of computational musculoskeletal modeling of scanned bone models (Charles et al., 2016; Regnault et al., 2021). However, its technical sophistication and the multivariate nature resulting from the IMMAs’ dependency on complex 3D joint poses over time complicate the analysis of IMMAs in more inclusive, phylogenetically broader datasets. Perhaps this is one reason why computational modeling has usually been used for in-depth analyses of the limb biomechanics of only one or just a few species (e.g., Hutchinson et al., 2005; Bates et al., 2012; Regnault and Pierce, 2018). Allen et al. (2021) were recently the first to analyze the IMMA in an explicitly phylogenetic comparative setting to allow evolutionary inferences about how changes in muscle topography (i.e., the attachment sites of the muscles on the bones) affect the IMMA of the pelvic muscles in 13 bird-line archosaur species. These authors ultimately averaged the IMMA values in their comparative analysis, hence not taking into consideration how the IMMA’s length varied with changes in a joint pose (Allen et al., 2021).

In contrast to the challenging determination of IMMAs, osteological muscle in-levers are typically used to provide information on musculoskeletal function in comparative analyses of ecomorphological specialization (see, e.g., Ward and Sussman 1979; Boyer et al., 2013). Osteological in-levers are defined as the distance between the attachment site of a muscle and the CoR of the joint the muscle is acting on (Hildebrand and Goslow, 1995). From a pragmatic standpoint, osteological in-levers can readily be analyzed using dry skeletal collection material and/or well-preserved fossil specimens not only in 2D (e.g., Vizcaino and Milne, 2002; Marshall et al., 2021) but also in 3D (e.g., Mielke et al., 2018; Wölfer et al., 2019), and without further knowledge of joint movements. The IMMA and the osteological in-lever are geometrically related. A larger osteological in-lever increases the distance between the MLoA and the CoR and hence will ultimately lead to an increase in all IMMAs. Thus, as is the case for the IMMAs, increasing in-lever length while keeping everything else constant results in a larger torque (Zajac, 1992; Payne et al., 2006; Moore et al., 2013; Marshall et al., 2021). Importantly, a longer muscle moment arm will also result in a decline in angular velocity at the joint given a constant contraction speed (Hutchinson et al., 2005; Channon et al., 2010). The osteological in-lever and the IMMA are exactly the same in a single joint pose, precisely when the in-lever is at a 90° angle to the MLoA (Figure 1A). Consequently, the in-lever also represents the largest possible IMMA. For all other poses, the IMMA is a penalized value of the in-lever length, that is, the in-lever length multiplied by the cosine of the angle between itself and the IMMA (a factor x with 0 ≤ x < 1; see equation in Figure 1A). This demonstrates that the in-lever alone misses important geometric information for the assessment of potential muscle torque. Its reliability to approximate the IMMA will depend on the functionally relevant joint range of motion in the taxa of interest.

Given the crucial role in musculoskeletal function, IMMAs can be expected to be under selective pressure and to reflect ecomorphological specialization (cf. Allen et al., 2021). In this study, we therefore aim at obtaining insight into how IMMA length changes reflect different functional demands specific to different locomotor lifestyles using a broad ecomorphological sample. Second, we will assess whether our methodologically more sophisticated IMMA analysis provides additional functional information that would be left unrecognized by utilizing a single, easily accessible, yet simplifying osteological proxy. In order to do so, we use musculoskeletal modeling of the lever-arm system associated with the M. gluteus medius and the broadest phylogenetic sample for IMMA analysis thus far, focusing on the Caviomorpha (Mammalia: Rodentia). The M. gluteus medius, a hip extensor muscle, has been demonstrated to be a main contributor to hip extension and partly to hip abduction during locomotion (e.g., Goslow et al., 1981; Larson and Stern, 2009). Also, the muscle shows clearly recognizable attachment sites on the pelvis and femur necessary to model the MLoA.

We chose the monophyletic and taxonomically diverse Caviomorpha (Fabre et al., 2012) because they exhibit a remarkable morphological disparity which was previously linked to the vast diversity of lifestyles and, specifically, locomotor behaviors (e.g., Elissamburu and Vizcaíno, 2004; Morgan, 2009; Morgan and Álvarez, 2013; Candela et al., 2017). Caviomorphs underwent radiation after the arrival of their most recent common ancestor in South America, which was isolated back then, starting more than 41 million years ago (mya) according to available fossil data (Pierre-Olivier et al., 2011). Today, the group is also distributed across the Caribbean islands and Central and North America (Wilson and Mittermeier, 2011). Four major lineages (Cavioidea, Erethizontoidae, Chinchilloidea, and Octodontoidea) are recognized, comprising about 250 extant species (Wilson and Mittermeier, 2011). Their ecological diversity encompasses subterranean/fossorial (e.g., tuco-tucos of the genus *Ctenomys*), semi-fossorial (e.g., the plains vizcacha *Lagostomus maximus*), semi-aquatic (e.g., nutrias of the genus *Myocastor* and capybaras of the genus *Hydrochoerus*), terrestrial (e.g., guinea pigs of the genus *Cavia*), cursorial (e.g., maras of the genus *Dolichotis*), ricochetal (*Chinchilla*), and scansorial/arboreal lifestyles (e.g., new world porcupines of the family Erethizontidae and hutias of the subfamily Capromyinae) (Nowak 1999; Wilson and Mittermeier, 2011). Caviomorpha also display a noticeable disparity in body size, with the smallest members of Octodontoidea being ∼50 g and the capybara weighing up to ∼50 kg (Wilson and Mittermeier, 2011).

**FIGURE 2:**
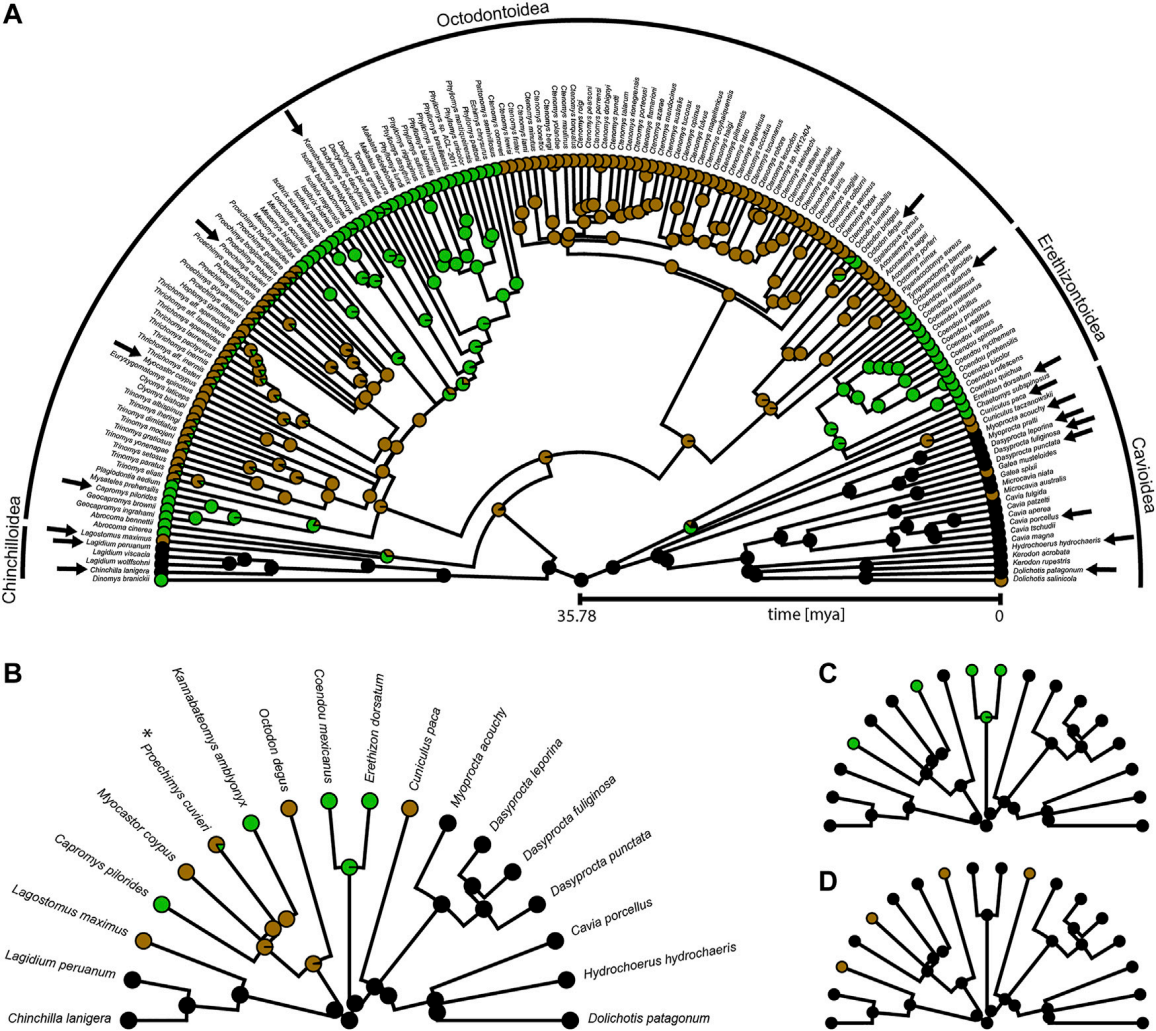
Phylogeny of Caviomorpha with reconstructed lifestyles. Pie charts at tips and nodes indicate posterior probabilities of lifestyles. **(A)** Original phylogeny containing all available species from Hedges et al. (2015). Cursorial category in black, fossorial category in brown, and scansorial category in green. Arrows indicate species included in our dataset. **(B)** Phylogeny and mapping from **(A)** after pruning to match our dataset for fitting an Ornstein-Uhlenbeck (OU) model with three optima (see text). Color coding as in **(A)**. Asterisk indicates the species that was used instead of *P. mincae* (see text). **(C)** Pruned phylogeny and mapping for fitting an OU model with two optima. Terrestrial category in black and scansorial category in green. The original mapping with all species before pruning is provided in Supplementary Figure S1A. **(D)** Pruned phylogeny and mapping for fitting an OU model with two optima. Overground category in black and fossorial category in brown. The original mapping with all species before pruning is provided in Supplementary Figure S1B.

We will use 18 caviomorph species representing the phylogenetic diversity of lineages within the Caviomorpha and categorize them into three locomotor categories: scansorial, cursorial, and fossorial (Figure 2). The former two categories can be considered to include all “overground species,” and the latter two categories comprise all “terrestrial species.” Generally, independent of lifestyle, we expect the maximum IMMA in the last third of possible extension, when maximum force is needed for powerful hip extension prior to lift-off for leaps or during the late stance in rapidly accelerating locomotor bursts (e.g., Aerts, 1998). We expect cursorial and scansorial (i.e., overground) species to emphasize powerful extension over the fossorial species because it is expected to be equally crucial for high-speed terrestrial locomotion, leaping, and climbing up an incline. However, cursorial and scansorial lifestyles should be reflected differently in terms of the M. gluteus medius IMMA; scansorial species can be expected to exhibit the largest IMMAs of our sample over a large portion of hip extension, as, for these species, maximum torque during climbing should be more important than fast joint excursions (which would be compromised by overly large IMMAs, see Allen et al., 2021). Cursorial species, on the other hand, might display some sort of compromise between large torque generation for fast acceleration and angular velocity for fast hip extension at maximum running speed. Fossorial caviomorphs dig with their forelimbs as most digging mammals (Hildebrand and Goslow, 1995; Nowak, 1999; Wilson and Mittermeier, 2011), but the role of the hind limbs is usually uncertain or not described. Caviomorphs of the genus *Ctenomys* also use their teeth to break up hard soil (Wilson and Mittermeier, 2011), but they were not considered in this study. Given this information, we do not expect to find adaptations of the IMMA for powerful hip extension in these species. As the studied fossorial species are not completely subterranean but search for food overground and must be able to quickly flee from predators, they might also share similarities in the IMMA length changes with the cursorial species. This might justify the hypothesis that they, together as a “terrestrial” category, are distinguishable from the scansorial species.

**FIGURE 3:**
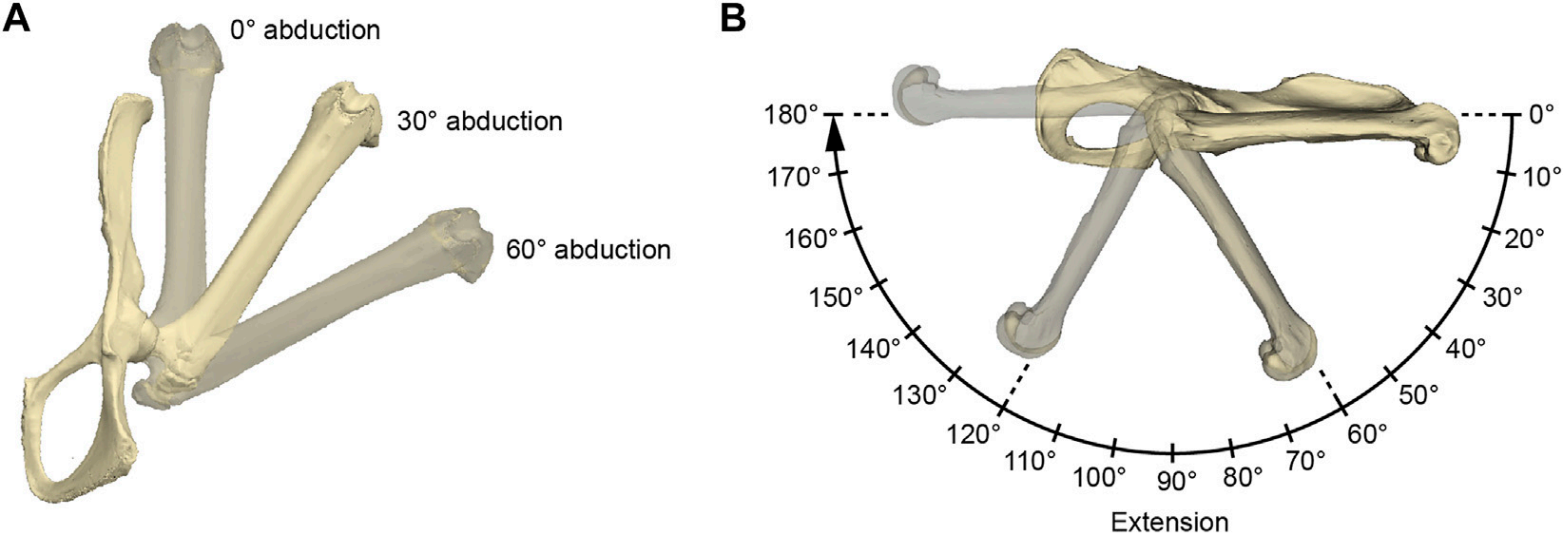
Illustration of the acquired hip joint angles. **(A)** Three abduction angles were used for each measurement series of extension angles **(B)**. The here depicted extension angle is 0°. **(B)** Procedure of a single measurement series. The 0° rotational angle in the 0° abduction setting as in **(A)** was used as the reference pose. The IMMAs were measured every 10° resulting in 19 values per abduction setting and 54 values per specimen in total. For further explanation, see text.

Our comparative functional modeling approach uses 3D bone models of the pelvis and the femur of each specimen to derive the IMMA from a simple musculoskeletal model while exploring the entire range of hip extension (i.e., femur retraction relative to the pelvis within the parasagittal plane) for three femoral abduction angles (Figure 3). Considering the IMMA over the entire range of motion throughout anatomically possible hip extension will allow a more nuanced evaluation of the functional implications for each locomotor category. In addition to locomotor lifestyle comparisons throughout hip extension *via* exploratory statistics, we will also apply macroevolutionary model comparison *via* likelihood evaluation (Butler and King, 2004) to selected joint poses. For this purpose, we will make use of the largest accessible caviomorph phylogeny to reliably reconstruct caviomorph lifestyle evolution. To compare the IMMA results with those of a simple proxy, the distal osteological in-lever of the M. gluteus medius relative to the hip joint will be measured as well for all species and similarly compared among locomotor categories.

## 2 Materials and Methods

### 2.1 Specimen Sampling

Femora and pelves were collected from a single specimen of 18 species in total housed in the collections of Naturhistorisches Museum Vienna (NMW), Austria, and Museum für Naturkunde Berlin (MfN), Germany (Supplementary Table S1). The bones were scanned with a surface laser scanner *via* the software “Scantools” (Kreon “Skiron” laser scanner for MicroScribe, Solution Technologies, Inc., Oella, United States) or, if too small for surface laser scanning, using a µCT scanner (Phoenix Nanotom M, General Electric, Boston, United States) with the software VGSTUDIO MAX (Volume Graphics, Heidelberg, Germany). Minor defects of the meshed surface models were repaired using the software MeshLab version 1.3.3 (Cignoni et al., 2008) and Geomagic Wrap 2017 (3D Systems, Rock Hill, United States).

### 2.2 Functional Modeling Approach of IMMAs

The functional modeling approach was conducted using Autodesk Maya 2019 (Autodesk, San Rafael, United States). Surface models of a specimen’s femur and pelvis were imported, and the same reference pose was created for each specimen in a separate scene by aligning its pelvis to the coordinate system of Autodesk Maya (Figure 3). The symphyseal surface (facies symphysialis) was used for the cranio-caudal orientation of the pelvis (Popesko et al., 1992). For all the specimens of which only one of the hip bones were scanned, a realistic degree of tilt had to be estimated. This was carried out by inclining the pubic bone’s inner surface to form a smooth parabolic transition (pictured from a cranial perspective) to the imagined contralateral pubic bone to which it is connected *via* the symphysis. However, scans originating from NMW collections mostly included at least the contralateral pubic bone and parts of the ischium, which made this estimation obsolete. The configuration in which the long-axis of the protracted femur and the cranio-caudal orientation of the pelvis were aligned in Autodesk Maya’s side view was defined as the extension angle of 0° (Figure 3). Similarly, the abduction angle of 0° was defined by the configuration when the long-axis of the protracted femur and the cranio-caudal orientation of the pelvis’s pubic symphysis were parallel in Autodesk Maya’s top view. Both abduction and extension angles being 0° were determined as the reference pose.

To model hip extension and to allow comparison of standardized IMMA measurements across all 18 species, the measurement series was conducted in three different setups of hip abduction for each model (Figure 3): 0° abduction (i.e., true parasagittal limb movement), 30° abduction, and 60° abduction. Starting from an extension angle of 0°, the extension was then modeled in 10° increments up to 180°. This resulted in 19 × 3 = 57 poses per specimen in which the IMMA was measured.

The IMMA in each pose was measured in the following way. A “locator” was positioned each at the muscle origin and the muscle insertion of the M. gluteus medius. A recognizable landmark of both attachment sites was chosen for each “locator” position to facilitate easy and replicable placements in all specimens. For the muscle origin, a landmark was defined at the center of the attachment of the M. gluteus medius’ deep part (Figure 1). The position of this attachment site can vary quite distinctly as, for example, it was shown to be the case in squirrels (Sokolov, 1964). Comparable publications providing muscle maps for various caviomorph rodents (especially those studied here) were not available to our knowledge, but a map for *Hydrochoerus hydrochaeris* was published by Garcia-Esponda and Candela (2010). Based on their drawings, we approximated the center of the M. gluteus medius origin within the center of the dorsal fossa of the outer wing of the ilium (Figure 1). For the muscle insertion, the trochanter major’s most proximal point was chosen. The functions of Autodesk Maya were used to visualize the line of action of the muscle connecting both “locators.” To simplify the models in this comparative study, it was decided to use a straight line of action crossing insertion and origin “locators” since the exact positioning of other surrounding muscles was mostly unknown in all 18 caviomorph species. Therefore, information for the implementation of “*via* points” and “wrapping surfaces” to guide more realistic muscle lines of action (Hutchinson et al., 2005) could not be provided. A sphere was fitted into the caput femoris and a new “locator” point-constrained into its center, now marking the CoR. Then, this “locator” position was set as a reference point to Maya’s “nearest point on curve” function using the node editor. Another “locator” was set as the function’s output, thereby visualizing the result as the projected point on the line of action. The projection could either fall on the MLoA or outside (extending the straight MLoA further beyond the “locators” marking the muscle attachment sites), depending on the degree of extension (Figures 1, 3). To finally obtain IMMA lengths, the distance between the CoR and the projected point on the line was measured using the “distance between” function.

Measurements in Maya units had to be converted to the metric system. To accomplish this, the femoral length and width of the condyle of the eight femora from the MfN collections in Berlin were measured using a ruler in centimeters and divided by the corresponding digital measurement of the length of the 3D bone model in Autodesk Maya (Maya units). Afterward, all 16 ratios (Supplementary Table S1) were averaged and all measurements in Maya were multiplied by this mean value (1.017). All length measurements in centimeters are provided in Supplementary Table S2.

### 2.3 Body Mass Proxy

We aimed at accounting for body mass in the subsequent analyses as it is a typical confounding factor compared to locomotor ecology. The body masses of the analyzed specimens were not available. Since Wölfer et al. (2019) showed that the anteroposterior diameter of the femoral midshaft (called just femoral diameter in the following) is strongly correlated to body mass and bears almost no lifestyle signal in the related rodent taxon Sciuromorpha, we used it here to approximate the effect of body mass. The femoral diameter was measured three times on each surface scan using Geomagic Wrap 2017 (3D Systems, Rock Hill, South Carolina, United States) and then averaged (Supplementary Table S3).

### 2.4 Graphical Comparison of Extension Poses Among Lifestyles

We used R version 4.1.2 (R Core Team, 2021) for this and all subsequent analyses. The packages “readxl” (Wickham and Bryan, 2019), “tidyverse” (Wickham et al., 2019), and “ggpubr” (Kassambara, 2020) were used for data preparation and visualization. For each of the three abduction angles, the mean and the standard error of the IMMA of each lifestyle category were plotted for all 19 extension angles for graphical comparison. To account for the effect of body mass on the IMMA, we divided all values of a species by its femoral diameter which we call the normalized IMMA in the following. The measurement series at 30° abduction was believed to be the most realistic representation of the actual utilized joint range due to limited bone collisions and similar values of hip abduction found in *in vivo* studies of small- to medium-sized mammals (Jenkins, 1971). Therefore, this abduction setting was focused on the subsequent macroevolutionary model comparison analysis.

### 2.5 Macroevolutionary Modeling Approach

Based on the graphical comparison of the lifestyles’ mean normalized IMMAs, we compared different macroevolutionary models *sensu* Butler and King (2004) for the IMMA of three different extension angles approximately referring to the early stance, mid-stance, and late stance of a limb contact during cyclic locomotion: 40°, 80°, and 120° (Fischer et al., 2002). We considered the Brownian motion (BM) model (Felsenstein, 1985; Grafen, 1989) and the Ornstein–Uhlenbeck (OU) model (Hansen, 1997) as plausible macroevolutionary models for IMMA evolution. We interpreted the BM model with its constant stochastic rate of change as a non-adaptive null model of phenotypic drift. The OU model was used to represent adaptive evolution in which selection acts on the trait. The strength of selection is determined by the adaptive rate α and the distance of the trait from a hypothetical primary (interspecific) optimum θ specific to a particular selective regime (lifestyle category in our case). In addition to selection, random perturbations on the trait are modeled with a rate σ, representing the joint effect of not only all non-adaptive factors but also adaptive factors with minor influence compared to the selective regime (Hansen, 1997). The expected trait value is a weighted sum of the ancestral state and the past optima a species evolved toward (Hansen, 1997). Kopperud et al. (2020) added the option to include a direct effect on the trait in the R package “slouch.” We used this option to include the femoral diameter of a species to model it as exerting an instantaneous effect on the mean IMMA of the species. The α and σ values were transformed into the phylogenetic half-life (the time it takes for a species on average to evolve halfway to the optimum) and stationary variance (interspecific variance that remains constant after a certain time depending on the relative strength of α and σ) (Hansen, 1997).

We here defined three OU models that we considered plausible from a morphofunctional standpoint. The first model contained three optima (OU3), one for each lifestyle category. Two further models with two optima (OU2) each were further defined. The first OU2 model assumed a shared optimum between cursorial and fossorial species (called the terrestrial category; OU2_terr_). The second OU2 model assumed a shared optimum between cursorial and scansorial species (called the overground category; OU2_over_).

For each of the three OU models, we proceeded as follows. The lifestyle categories were reconstructed for macroevolutionary modeling using stochastic character mapping (Huelsenbeck et al., 2003) and the R packages “geiger” (Pennell et al., 2014) and “phytools” (Revell, 2012). We took the phylogeny from Hedges et al. (2015) which was pruned to an ultrametric tree of 169 caviomorph species (Figure 2). Lifestyle information was obtained from species descriptions in Wilson and Mittermeier (2011) and Nowak (1999) (Supplementary Table S4). Using the OU3 model as a guideline, we characterized each species by the lifestyle that was described as most dominant, choosing among the three categories: cursorial (specialized in running and foraging overground), fossorial (digging burrows and foraging overground), and scansorial (climbing trees and bushes, but perhaps also traveling on the ground). For many species or genera, lifestyle information was either not available, ambiguous, or a mixture of our categories. Hence, to include uncertainty for those tips, we decided to define the prior probability of each of the three lifestyle categories on the basis of the lifestyles of the closest relatives (Supplementary Table S4). In the case of the OU2 models, the prior probabilities of the two lifestyles now collapsed into a single lifestyle were simply added up. All transition rates among lifestyle categories were assumed to be different, and 1000 character maps were generated using the “make.simmap” function of the package “phytools” (Figure 2; Supplementary Figure S1).

We then trimmed all 1000 phylogenies including their stochastic character maps using the “drop.tip.simmap” function of the package “phytools” to match the species of our dataset. For this purpose, we substituted the name of the sampled species *Proechimys mincae* with the name *P. cuvieri*, as the former was not represented in the phylogeny. This was justified by the fact that it constituted the only sampled species of this genus. We then used the likelihood distribution of lifestyle categories at each node to determine the most likely lifestyle at each node. The branches were then assigned the most likely lifestyle of the preceding node using the “slouch.fit” function of the package “slouch” as part of the model fitting process (Supplementary Figure S2).

For model fitting, the IMMA and the femoral diameter were natural-log-transformed. The “slouch.fit” function works interactively by manually defining a range of half-lives and stationary variances of which all combinations are assessed in terms of their likelihood. The maximum likelihood combination was used to compute all regression statistics, but the two-unit support regions (i.e., the minimum and maximum values that were recovered two log-likelihood below the maximum likelihood) of the half-life and the stationary variance were provided as well as the standard errors for the optima and slopes. The likelihood of the four models was compared using the Akaike information criterion corrected for small sample sizes (AICc) and Schwarz information criterion (SIC), which penalize the maximum likelihood according to model complexity (see Burnham and Anderson, 2002).

### 2.6 Analysis of Osteological In-Lever

The lengths of the osteological in-levers of the femur (Supplementary Table S3) were obtained using the “distance measurement tool” in Maya. The osteological in-lever was defined as the distance from the insertion of the M. gluteus medius to the CoR. The macroevolutionary modeling procedure explained earlier for the IMMAs of the three selected joint poses was also applied to the in-lever data to compare their results.

## 3 Results

### 3.1 Convergent Evolution of Scansorial and Fossorial Lifestyles From a Cursorial Common Ancestor

The cursorial lifestyle was the most likely state at the root of the phylogeny (Figure 2). The fossorial lifestyle evolved six times independently, always from a cursorial ancestor within Caviomorpha. The scansorial lifestyle was most likely acquired five times independently, twice from a cursorial ancestor and three times from a fossorial ancestor.

### 3.2 Differences in IMMAs Among Lifestyles Depend on Extension Angle

**FIGURE 4:**
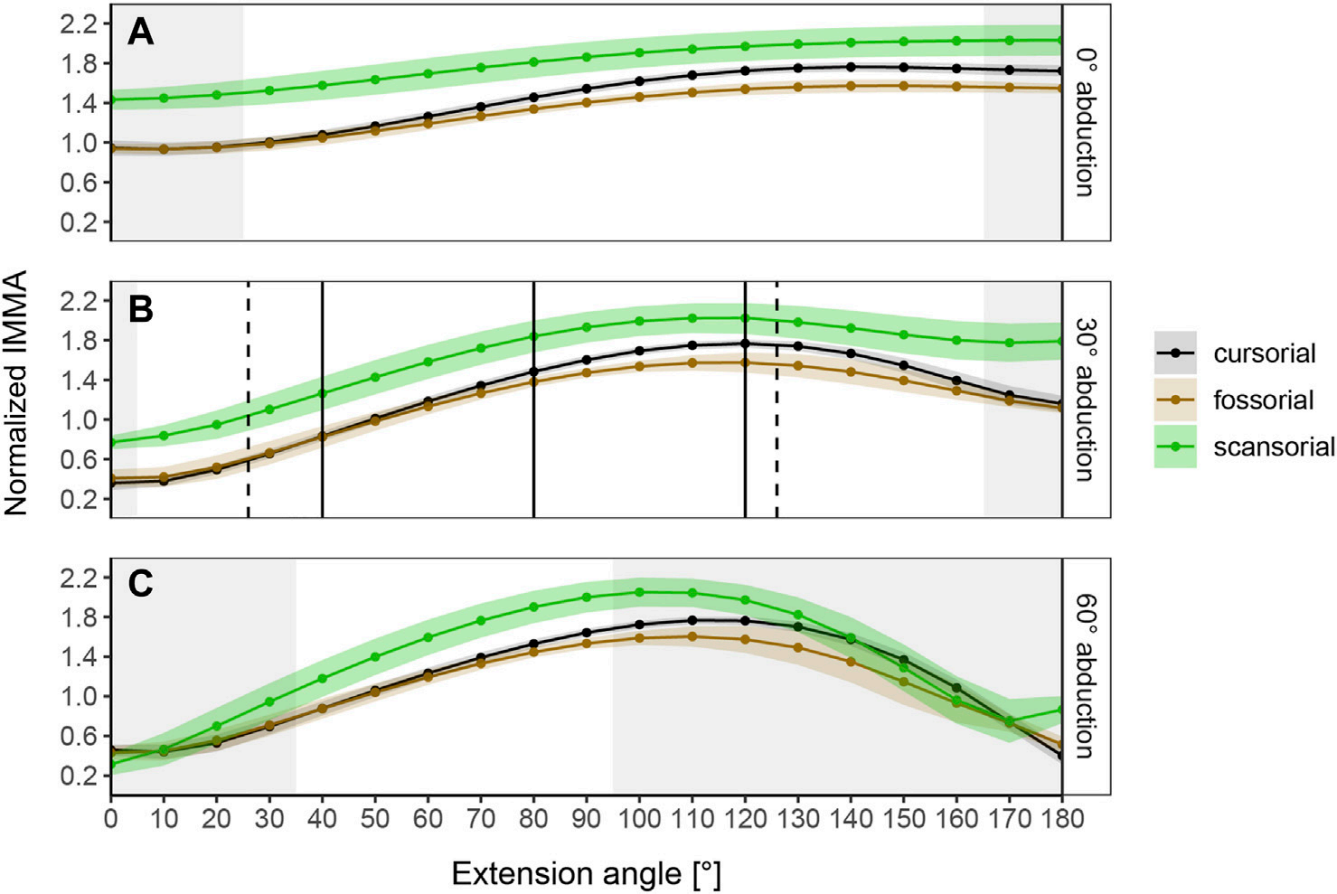
Curve progressions of normalized IMMA lengths averaged (±S.E.) per lifestyle category. The normalized IMMA was obtained by dividing the IMMA [cm] by the anteroposterior diameter of the femoral midshaft of the species. Gray areas depict extension angles that are anatomically impossible due to bone collisions in at least one specimen of the dataset. **(A)** Measurement series at 0° abduction. **(B)** Measurement series at 30° abduction. Solid black vertical lines mark the three extension angles (40°, 80°, and 120°) used for macroevolutionary modeling. Dashed vertical lines mark the touchdown (26°) and lift-off (126°) extension angles of the small caviomorph rodent *G. musteloides* (*in vivo* data from Fischer et al., 2002). **(C)** Measurement series at 60° abduction.

The normalized IMMA measurements averaged per locomotor category show different sigmoidal curve progressions throughout extension while lifestyle differences themselves depend on the abduction angle (Figure 4). At 0° abduction, the curves start with a comparatively large normalized IMMA value and have a rather shallow slope until a plateau is reached at around 130° hip extension. At 30° and 60° abduction, the curves start with lower values, reaching a similar peak as during 0° abduction, but a bit earlier at an extension angle around 100–120°. In comparison to 0° abduction, the curves tend to fall off again in model setups of 30° and 60° abduction. This effect is larger at 60° than at 30° abduction. In the range of extension angles that are anatomically possible (as judged by bone collisions during modeling in Maya) among all species, scansorial species always show a larger mean normalized IMMA length than the other lifestyle categories. Cursorial species tend to be similar on average to the fossorial species at smaller extension angles but fall in between them and the scansorial species at larger extension angles, resulting in the slightly steeper slope of their mean IMMA curve progression (Figure 4). The scansorial species generally display the largest standard error, which might be due to their lowest sample size, but also due to their larger variability (Supplementary Figure S3).

The range of anatomically possible extension angles is widest at 30° abduction, slightly reduced at low extension angles of 0° abduction, and drastically narrowed down at 60° abduction (Figure 4). Notably, at 30° abduction, which we believed to be the most plausible joint range for caviomorph locomotion, we observed the specimen of the fossorial species *O. degus* to display its peak IMMA at a smaller extension angle of 80° compared to other fossorial species (Supplementary Figure S3).

### 3.3 Most Likely Macroevolutionary Model Depends on Extension Angle

The regression models revealed that all IMMAs scale positively with the femoral diameter (Table 2), justifying the normalization. The IMMA at 40° extension scaled with negative allometry (b = ∼0.8), whereas the other two IMMAs at 80° and 120°, respectively, scaled close to isometry (b = ∼1).

**FIGURE 5:**
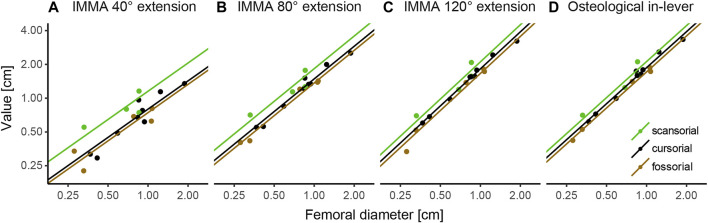
Regression plots of instantaneous muscle moment arms (IMMAs) and osteological in-lever vs. femoral diameter. IMMAs were taken from three different extension angles **(A–C)** at 30° abduction. **(D)** Osteological in-lever. Lines indicate the regression slopes with varying intercepts (i.e., the primary optima for a given femoral diameter) among lifestyle categories as estimated by the most likely macroevolutionary model (Table 1). The coordinate axes were log-transformed.

According to the AICc, which was preferred over SIC to rank the models, the OU2_terr_ model is always the most likely one, followed by the OU3 model (Table 1). The SIC values show only minor discrepancies and an overall similar ranking. The likelihood difference of the remaining models was large enough to consider them comparably implausible. The OU2_terr_ model always estimated a larger optimal value for the IMMA of the scansorial group compared to that of the terrestrial group (the combination of fossorial and cursorial species; Table 2). In all OU3 models, the optimal IMMA values for a given femoral diameter were estimated to decrease from scansorial to cursorial and finally fossorial species (Table 2; Figure 5). Overall, this supported our exploratory findings; however, Figure 4 suggests that fossorial and cursorial species share a similar mean IMMA at 40° extension. This discrepancy between the mean normalized IMMA and the IMMA optima of these two groups at 40° extension might result from the fact that normalization was executed using femoral diameter, whereas a regression exponent of 0.8 instead of 1 was recovered (Table 2). The AICc difference between the OU2_terr_ and the OU3 model decreased with increasing extension angle (Table 1).

**TABLE 1:** Fitted macroevolutionary models ranked according to Akaike information criterion (AICc) in ascending order.

IMMA 40° extension			IMMA 80° extension			IMMA 120° extension			Osteological in-lever		
Model	SIC	AICc	Model	SIC	AICc	Model	SIC	AICc	Model	SIC	AICc
OU2_terr_	5.52	6.07	OU2_terr_	−15.69	−15.14	OU2_terr_	−17.97	−17.42	OU2_terr_	−22.03	−21.48
OU3	7.96	10.26	OU3	−14.71	−12.42	OU3	−18.07	−15.77	OU3	−22.47	−20.18
BM1	12.39	11.43	OU2_over_	−9.5	−8.96	OU2_over_	−15.01	−14.46	OU2_over_	−19.81	−19.26
OU2_over_	12.81	13.36	BM1	−7.4	−8.36	BM1	−11.23	−12.19	BM1	−12.75	−13.71

IMMA, instantaneous muscle moment arm; SIC, Schwarz information criterion.

**TABLE 2:** Summary statistics of the two most likely macroevolutionary models per trait (Table 1).

Model	θ ± S.E.	b ± S.E.	hl (ML {2-USR})	v_y_ (ML {2-USR})
IMMA 40° extension				
OU2_terr_	Scansorial: 0.14 ± 0.1 Terrestrial: −0.26 ± 0.06	0.81 ± 0.09	≤0.001{∼0; 36.51}	0.04 {0.02; 0.08}
OU3	Cursorial: −0.23 ± 0.06 Fossorial: −0.31 ± 0.09 Scansorial: 0.13 ± 0.1	0.84 ± 0.09	≤0.001{∼0; 38.39}	0.03 {0.02; 0.108}
IMMA 80° extension				
OU2_terr_	Scansorial: 0.6 ± 0.06 Terrestrial: 0.36 ± 0.03	0.98 ± 0.05	4.03 {∼0; 39.6}	0.01 {0.01; 0.03}
OU3	Cursorial: 0.38 ± 0.04 Fossorial: 0.3 ± 0.05 Scansorial: 0.59 ± 0.06	0.97 ± 0.05	4.56 {∼0; 32.48}	0.01 {0.01; 0.02}
IMMA 120° extension				
OU2_terr_	Scansorial: 0.75 ± 0.05 Terrestrial: 0.56 ± 0.03	1.12 ± 0.05	≤0.001 {∼0; 31.01}	0.01 {0.01; 0.02}
OU3	Cursorial: 0.59 ± 0.03 Fossorial: 0.5 ± 0.05 Scansorial: 0.74 ± 0.05	1.1 ± 0.04	≤0.001 {∼0; 21.11}	0.01 {<0.01; 0.02}
Osteological in-lever				
OU2_terr_	Scansorial: 0.76 ± 0.05 Terrestrial: 0.6 ± 0.03	1.08 ± 0.04	2.42 {∼0; 17.85}	0.01 {<0.01; 0.02}
OU3	Cursorial: 0.63 ± 0.03 Fossorial: 0.54 ± 0.04 Scansorial: 0.75 ± 0.04	1.07 ± 0.04	0.12 {∼0; 13.24}	0.01 {<0.01; 0.01}

2-USR, two-unit support region; b, direct regression coefficient for the effect of the anteroposterior diameter of the femoral midshaft; hl, phylogenetic half-life; IMMA, instantaneous muscle moment arm; ML, maximum likelihood estimate; θ, optima at zero diameter, that is, regression intercepts; v_y_, stationary variance.

For the three extension angles, these two most likely models (but also all other fitted models) recovered a very small phylogenetic half-life and also a small stationary variance, indicating the relatively strong selective pressure induced by lifestyle and relatively minor influences of other evolutionary factors. The maximum likelihood estimate of the phylogenetic half-life was usually less than 1000 years for the extension angles of 40° and 120°, and from 4 to 4.6 million years (my) for the angle of 80°. This suggests almost instantaneous adaptation since the root of Caviomorpha was dated 35.78 mya and all investigated lineages evolved in their lifestyle longer than 15 my (Figure 2). However, the maximum likelihood values have to be interpreted with caution, as the two-unit support regions suggest plausible half-lives of up to ∼40 my for the most likely models (Table 2). In this extreme case, a caviomorph lineage from the root to the present would have only evolved on average about halfway to its optimum.

#### 3.3.1 Comparison Between In-Lever and IMMAs

The model ranking of the osteological in-lever according to AICc was most similar to that of the IMMA at an extension angle of 120° (Table 1), which becomes also apparent from the similarity of intercepts in the regression plots of Figure 5. This seems plausible as the peaks of the average IMMA curve progressions were observed around this extension angle, and thus, the IMMA values here were the closest to the osteological in-lever.

## 4 Discussion

In this study, we modeled the IMMAs of the M. gluteus medius, a primary hip extensor, over the entire range of anatomically possible hip extension in a phylogenetically informed comparative framework. The general assumption of selective pressure shaping muscle moment arms was tested using a comparison of moment arms in a broad sample of caviomorph rodents with diverse locomotor specializations. Although many studies investigated moment arms in diverse tetrapod muscles, a comparison between existing studies is not easily made due to differences in methodologies (e.g., Lieber and Boakes, 1988; Payne et al., 2006; MacFadden and Brown, 2007; Channon et al., 2010; Charles et al., 2016). Only very few studies looked into instantaneous moment arms from a phylogenetic point of view (e.g., Allen et al., 2021). We will first discuss general IMMA patterns that affect the torque generation of the M. gluteus medius at the hip joint, then proceed to lifestyle-specific findings, then compare our IMMA results to the osteological in-lever results, and finally discuss limitations and future directions.

### 4.1 M. Gluteus Medius IMMA Benefits Large Extension Angles

Our modeling of IMMAs revealed that caviomorph species, regardless of locomotor category, display a sigmoidal change in the M. gluteus medius’ IMMA with an increase of the extension angle relative to the pelvis, independent of each tested abduction angle (Figure 4). Perhaps, this is a consequence of an overall relatively consistent anatomical geometry of the hip across all Caviomorpha (and perhaps Mammalia) which presumably cannot be dramatically altered without deleterious effects on other functionalities of the hind limb locomotor system. All species (except for *O. degus*) exhibit large IMMAs at extension angles that can be expected to occur at the late stance phase (>90°; cf. Rocha-Barbosa et al., 1996; Fischer et al., 2002). This might benefit fore-aft acceleration, which usually occurs during the second half of the stem phase (Witte et al., 2002; Lammers et al., 2006; Zumwalt et al., 2006; Walter and Carrier, 2009; Hesse et al., 2015; Granatosky et al., 2020; Wölfer et al., 2021). However, the hip joint angle does not necessarily increase constantly during the stem phase, but its length change could depend on the gait. For example, a study by Rocha-Barbosa et al. (2005) on the limb kinematics of the guinea pig *Cavia porcellus* demonstrated that during trot—a symmetrical gait usually used at moderate running speeds (Hildebrand and Goslow, 1995)—the mean extension angles at touchdown, mid-stance, and lift-off were 58°, 67°, and 115°, respectively, indicating constant hip extension. In the modeling approach of our work, the M. gluteus medius IMMA of *C. porcellus* steadily increased in this range, exemplifying the benefit explained previously. However, during gallop—a synchronous gait usually used at high running speeds (Hildebrand, 1977)—the mean extension angles at the three time points were 91°, 85°, and 116°, respectively (Rocha-Barbosa et al., 2005). This suggests no constant extension, but a retention or even slight flexion during the first half of the stem phase, which could also be observed for other small-sized mammals (see Fischer et al., 2002). In this case, initiating stance with an already large extension angle relative to the pelvis and maintaining that angle during the early stance might assist positive torque generation (promoting hip extension) to counter the negative torque (promoting hip flexion) that is generated by the center of mass of the body on the hip joint as this center is most likely located in front of the hind limbs. On the contrary, in symmetrical running gaits such as the trot, this might not be necessary, as one fore- and one hind limb both simultaneously touch the ground to support the center of mass in between them (Hildebrand, 1985).

Not only the hip joint kinematics were found to be variable in small mammals, but also their net hip torque patterns, with different curve progressions and net torque maxima occurring at different times during stance, which could be partly linked to the preferred gait of the species (Witte et al., 2002). For example, Witte et al. (2002) observed the caviomorph species *Galea musteloides* to constantly increase the net positive hip joint torque and hip extension throughout stance while using symmetrical gaits, supporting the benefit of powerful acceleration during late stance. However, another species (*Tupaia glis*, belonging to the mammalian order Scandentia) primarily characterized by synchronous gaits first displayed a negative hip joint torque associated with hip flexion at the beginning of stance (Witte et al., 2002). This would support the additional need for high muscle-generated torques in the hip during early stance to prevent even higher net negative torques that would collapse the hip joint. Thus, gait choice is also a critical confounding factor that needs to be considered when discussing differences among lifestyles.

### 4.2 Functional Significance of M. Gluteus Medius IMMAs for Different Lifestyles

The most likely low phylogenetic half-life that we always recovered for the most likely models suggests the IMMA to be functionally significant throughout hip extension and under selective pressure from the considered lifestyles. Despite the overall similarities in normalized IMMA curve progressions, our results indicate that the geometry of the hip does not strictly constrain IMMA adjustments but allows for independent adjustments at different extension angles. This is reflected, for example, in the diversity of the shapes of the sigmoidal IMMA curve progression among species (e.g., Supplementary Figure S3). But it is also visible in the fact that differences in the average IMMA between fossorial and cursorial species increased with increasing extension angle (Figure 4).

In general, our expectations based on considerations of the differential functional demands associated with cursorial, scansorial, and fossorial lifestyles in caviomorphs were met by our results. We documented larger M. gluteus medius IMMAs on average in scansorial caviomorphs in comparison with fossorial and cursorial species, which were more similar. This was also supported by the OU2_terr_ model always ranking the highest with scansorial species displaying the largest optimum. At larger extension angles, cursorial caviomorph species fell in between fossorial and scansorial species, reflected also in the OU3 model becoming more likely, perhaps because this phase is most important for acceleration, as hypothesized, however, apparently only at larger extension angles.

The cursorial lifestyle was found to be the ancestral state in Caviomorpha. We expected to find a trade-off in the IMMAs between the capacity for the generation of hip extensor torque and faster hip extension. This was based on previous analyses of the geometrically related osteological in-lever. Smith and Savage (1956), for example, argued that the gluteal muscle complex of cursorial mammals should be specialized for high angular velocity, which would imply that a relatively short trochanter and, thus, short IMMAs are beneficial. On the other hand, a relatively long trochanter major and thus gluteal muscle osteological in-lever had previously also been associated with cursorial locomotion (Polly, 2007; Croft and Anderson, 2008). Interestingly, we observed particularly shorter M. gluteus medius IMMAs in our models at little hip extension in cursorial species when compared to scansorial species (but not when compared to fossorial species). This indicates an optimization for increased angular velocity during little hip extension. However, another explanation for a relatively small IMMA could be that especially small cursorial species are expected to exploit fast synchronous gaits, for example, to escape predators (Hildebrand, 1977). As discussed earlier, the stem-phase during synchronous gaits might be initiated by already fairly large extension angles, perhaps mitigating the selective pressure on high torques for fast accelerations at smaller extension angles. Closer to the maximum anatomically possible hip extension of cursorial species (i.e., at values that can be expected during the late stance of Caviomorpha; cf. Rocha-Barbosa et al., 1996; Fischer et al., 2002), the gluteal IMMAs appeared to fall on average between those of scansorial and fossorial species, respectively. This might hint at the trade-off between torque generation and angular acceleration in the hip of cursorial species mentioned earlier.

We found that the scansorial lifestyle is a derived condition within Caviomorpha. Scansorial locomotion requires powerful hip extension during climbing and locomotion on inclines when a larger proportion of the body’s mass rests on the hind limbs and additionally needs to be pushed upward against gravity (e.g., Preuschoft, 2002; Lammers et al., 2006; Hesse et al., 2015; Wölfer et al., 2021). Particularly, Channon et al. (2010) described strong hip extension by gluteal muscles as beneficial for the highly arboreal locomotion of gibbons. A similar adaptation of hip extensors would be necessary to facilitate powerful hip extension during launches for leaps from one support to the next in the discontinuous arboreal habitat of scansorial species (Aerts, 1998; Scholz et al., 2006). Indeed, the optimal IMMAs of the scansorial caviomorphs always scored as the largest optima, and this trend was also retrieved graphically after normalization with femoral diameter. These findings agree with those of Wölfer et al. (2019) who investigated the osteological in-lever of the M. gluteus medius in Sciuromorpha (squirrel-related rodents) and also found tree-dwelling species to display longer in-levers on average compared to fossorial/terrestrial species (though particularly for species of larger body masses). The outstandingly large IMMA optimum at the extension angle of 40° (1.5 times larger than that of the terrestrial species) suggests an importance of powerful torques at early stance. This coincides with the fact that scansorial species typically exploit symmetrical gaits with small extension angles during early stance (e.g., Biknevicious et al., 2013; Hesse et al., 2015; Karantanis et al., 2017). Additionally, independent of gait, it might compromise the crouched posture that is usually utilized during climbing on narrow substrates to bring the center of mass closer to the substrate and thus avoid large fatal toppling moment (Nakano, 2002; Schmidt and Fischer, 2011).

The fossorial lifestyle is also most likely a derived condition within Caviomorpha. As expected, the fossorial species of our study do not seem to rely as much on powerful hip extension on average. This is reflected in smaller IMMA optima at larger extension angles (80° and 120°) with respect to the ancestral cursorial condition. However, it is unclear, why, for example, the optimum at 40° extension (and thus in general for smaller angles) was retained and not reduced during evolution. We assume that relatively fast locomotion is still under selection for the species included in this study as they forage above ground (Wilson and Mittermeier, 2011) and should experience similar predation pressure as the remaining species in our study. However, this again cannot explain the overall reduced optima at larger extension angles. The reduced potential for powerful hip extension appears to be consistent with the primary use of the forelimbs to break loose hard soil during digging activities (Wilson and Mittermeier, 2011) and would suggest the forelimbs are adapted to powerful retractions. However, the scapula and the humerus of caviomorph rodents are mostly characterized by a phylogenetic instead of a functional signal (Morgan 2009; Morgan and Álvarez, 2013). Perhaps, an IMMA analysis of the forelimb retractors reveals a functional signal that is not present in the bone morphology as shown here. Additionally, contradicting our results, Wilson and Geiger (2015) compared femoral indices among caviomorph rodents with different lifestyles and concluded that the potential force-output of the M. gluteus medius should be largest in fossorial caviomorphs. This discrepancy might be a consequence either of different species samples, different measurements (they used a proxy for the in-lever), or different normalization approaches (they used the distance between proximal and distal femoral condyles, whereas we used femoral midshaft diameter).

### 4.3 IMMA Length Changes Are Preferable Over Osteological In-Lever due to Nuanced Insights Into Torque Optimization

Evolutionary model comparison of osteological M. gluteus medius in-levers of the analyzed specimens almost perfectly reproduced the results we obtained for the IMMA at 120° of modeled hip extension. This is plausible because this extension angle was characterized by the largest average IMMA of each lifestyle category (Figure 4), which thus is closest to the length of the osteological in-lever (Figure 1A). It indicates that for the hip joint, the in-lever only approximates the IMMA well at large extension angles. However, it failed to detect the similarity between cursorial and fossorial species and the pronounced dissimilarity between the former and scansorial species at lower values of hip extension, as suggested by the OU2_terr_ model. As shown earlier, this might hint at similar selective pressures from cursorial and fossorial locomotion as opposed to larger extension angles. On the other hand, the even more increased moment arm lengths in the early stance phase appear to present a significant adaptation of scansorial species to climbing on inclines. Therefore, high extensor torques are probably still achievable in the relatively little extended hip joint of scansorial species climbing in a crouched posture, which is not detectable from an osteological analysis alone. The realization of large IMMAs in this case is most likely only possible due to a position of the origin of the M. gluteus medius relatively farther from the hip joint. Smith and Savage (1956) pointed to pelvis morphology reflecting a specialization for the rapid extension of the thigh (as expected for cursorial species) or slower, but more powerful extension (as expected for scansorial species). In our dataset, we indeed observed considerable shape differences among pelves, especially concerning the wing of ilium, which most certainly affects the position of the attachment site for the M. gluteus medius. A broader quantitative study of caviomorph pelvis shape is necessary to confirm this.

### 4.4 IMMA Measurements Are Only a First Step to Capture the Dynamics of Lever-Arm Systems

Importantly, the comparative analysis of IMMAs is not the be all and end all of musculoskeletal function. Here, we have focused on one of many parameters that determine the functioning of a lever-arm system, but selection will ultimately act on this system as an integrated whole. For example, our results therefore do not necessarily mean that caviomorph rodents associated with the scansorial locomotor category are in fact generating a comparatively more powerful torque *in vivo*, since the muscle properties of every specimen could not be investigated in this comparative modeling approach and remain unknown (Channon et al., 2010; Charles et al., 2016). For instance, despite relatively small IMMAs at small extension angles, it may be possible for cursorial caviomorph rodents to generate a more powerful muscle moment than their fossorial counterparts simply by generating a larger muscle force, for example, through a larger cross-sectional area or a higher degree of muscle activation (Crook et al., 2010). Relatedly, a study that investigated muscle architectural parameters in the hind limb extensors of a striding and a jumping caviomorph rodent found such differences in muscle properties (Rosin and Nyakatura, 2017). The observed differences in the shape of the wing of ilium of the pelvis mentioned earlier could point at differences in muscle size that most likely are associated with a different potential for torque generation.

Another aspect that was not considered in this study is the out-lever, that is, the distance from the CoR to the point where the lever-arm system exerts a force on its environment (e.g., the autopodia; Hildebrand and Goslow, 1995; Smith and Savage, 1956). Cursorial species, for example, are often characterized by a relatively small mechanical advantage (i.e., the in-lever to out-lever ratio) of the M. gluteus medius, which is interpreted to be beneficial for fast limb rotation but disadvantageous for torque generation (Smith and Savage, 1956). However, these out-levers are usually measured on a single limb configuration with particular predefined angles for all joints distal to the lever-arm system of interest. This disregards the dynamic change of all joint angles during a stride cycle (see Fischer et al., 2002). Previous studies have already incorporated the dynamical change of the out-lever of specific muscles and highlighted the functional importance of dynamic gearing (change of the mechanical advantage) during the stem phase (e.g., Carrier et al., 1994; Carrier et al., 1998). Thus, in order to gain a deeper insight into the adaptability of these dynamics to different lifestyles, the next crucial step would be to simultaneously model the IMMA and the corresponding instantaneous out-lever.

Finally, our IMMA measurements only considered the M. gluteus medius, an important but not the only hip extensor of quadrupedal mammals. As the selection is assumed to act on the joint force-output of all extensor muscles, integrating other contributing pelvic muscles potentially adds additional nuance to the results in this current work (Hoy et al., 1988; Allen et al., 2021). Nevertheless, our phylogenetically informed analysis underscores that IMMAs of single muscular components can already be informative with regard to the functional demands selective regimes impose during evolution.

### 4.5 Current Limitations

The modeling of IMMA length changes presented here relied on a virtual abstraction of the hip extension process. We focused on hip extension along the parasagittal plane at three separately predefined abduction angles associated with the transversal plane. We believed one of these abduction angles (30°) to be the most plausible for locomotion. These simplified the actual *in vivo* hip joint movements which most likely combine hip extension with changes in hip abduction and hip adduction. Perhaps, femoral long-axis rotation plays a certain role (Nyakatura and Fischer, 2010; Fischer et al., 2018) and even translations could be considered. Methods to assess all six degrees of freedom in *in vivo* joint movement exist (e.g., Brainerd et al., 2010), but as of yet, neither large-scale nor exemplary comparative analyses on rodents as studied herein have been conducted to our knowledge. Furthermore, compromises had to be made for modeling the MLoA. The MLoA modeled here without considering individual muscle architecture benefits a simplified approach for comparative analysis of many specimens (18 in our case), but this comes at the expense of more realistic individual results that a specifically adjusted line of action with “*via* points” and “wrapping objects” would allow (cf., Hutchinson et al., 2005; Bishop et al., 2021; Demuth et al., 2022).

The approach that we undertook is also laden with certain statistical caveats that need to be tackled in the future. Functional modeling of IMMAs in a virtual environment is still time-consuming and most likely the reason why it has regularly only been applied to a single or few species (e.g., Channon et al., 2010; Regnault and Pierce, 2018) or using averaged values when analyzed within a phylogenetic framework (Allen et al., 2021). Although in this study a comparatively large interspecific IMMA dataset was used to explore lifestyle adaptations, only a single specimen per species was involved. We had to assume that the sampled specimen is close to the species mean and that the interspecific variability exceeds the intraspecific variability. These caveats also limited the interspecific sample size and rendered it necessary to narrow down the diverse locomotor ecologies in our study to just three relatively course locomotor categories. Thus, eventual anatomical differences existing between locomotor behaviors within a category (e.g., between ricochetal and striding cursorial caviomorphs; Rosin and Nyakatura, 2017) were not possible to identify in our comparative modeling approach. This may have resulted in less striking mean results per locomotor category in favor of a general overview. Finally, the limited number of species also hindered the use of more complex multivariate statistical analyses that are necessary to account for the correlation among IMMAs of different joint poses. Establishing automation algorithms for the functional modeling procedure could provide the opportunity to overcome these limitations.

## 5 Conclusion

Taken together, the current study demonstrates nuanced insights into how the functional significance of instantaneous M. gluteus medius moment arms differs between locomotor lifestyles of caviomorph rodents. It also highlights that ecomorphological specializations at smaller hip extension angles could not have been identified by the use of the simple osteological in-lever. Our findings thus underpin the importance of functional modeling for the understanding of the adaptive significance of lever-arm systems. However, future studies on other joint systems are needed to assess its general importance and in which cases osteological measurements can still be sufficient. This is especially relevant for fossils that often lack skeletal elements and, thus, potentially essential lever-arm components for functional modeling.

Several key limitations to our modeling approach are discussed. Despite new functional and evolutionary insights, a future investigation of other aspects of musculoskeletal function (such as muscle activation patterns, muscle architecture, and muscle fiber type composition) would be necessary for a comprehensive understanding of the implications of moment arm values on actual joint torque, as multiple studies have pointed out (e.g., MacFadden and Brown, 2007). This should be accompanied by the simultaneous study of instantaneous out-levers to the point of contact to the support to obtain a deeper understanding of the dynamics of force-output throughout the locomotor cycle. Furthermore, for a more comprehensive understanding of how locomotion affects hind limb retraction in caviomorph rodents, not only other gluteal muscles should be subjected to a comparative modeling approach but also muscles of the ischiopubic complex, such as the M. semimembranosus and the M. biceps femoris (Smith and Savage, 1956).

## Data Availability

The original contributions presented in the study are included in the article/Supplementary Material, further inquiries can be directed to the corresponding author. Additionaly, the related Maya files for the assessment of the instantaneous muscle moment arms of all 18 species are made available in an open access data repository (Löffler et al., 2022).
