# Supplementary material for: Computational Modeling of Gluteus Medius Muscle Moment Arm in Caviomorph Rodents Reveals Ecomorphological Specializations
Source: Front Bioeng Biotechnol. 2022 May 25;10:806314. doi: 10.3389/fbioe.2022.806314 (PMC9174681; doi:10.3389/fbioe.2022.806314)
Supplement: Supplementary file 1 [file DataSheet1.zip › Löffler et al. supplementary figures.docx]

Computational modelling of gluteus medius muscle moment arm in caviomorph rodents reveals ecomorphological specializations

Lukas Löffler^†^, Jan Wölfer^†^, Flavia Gavrilei, John A. Nyakatura*

AG Vergleichende Zoologie, Institut für Biologie, Humboldt-Universität zu Berlin, Philippstraße 13, 10115 Berlin, Germany

^†^: These authors have contributed equally to this work.

*: Corresponding author: john.nyakatura@hu-berlin.de

Keywords: moment arms, locomotion, modelling, hind limb, Caviomorpha, hip joint

Short title for headers: Moment arms of M. gluteus medius in caviomorph rodents

**
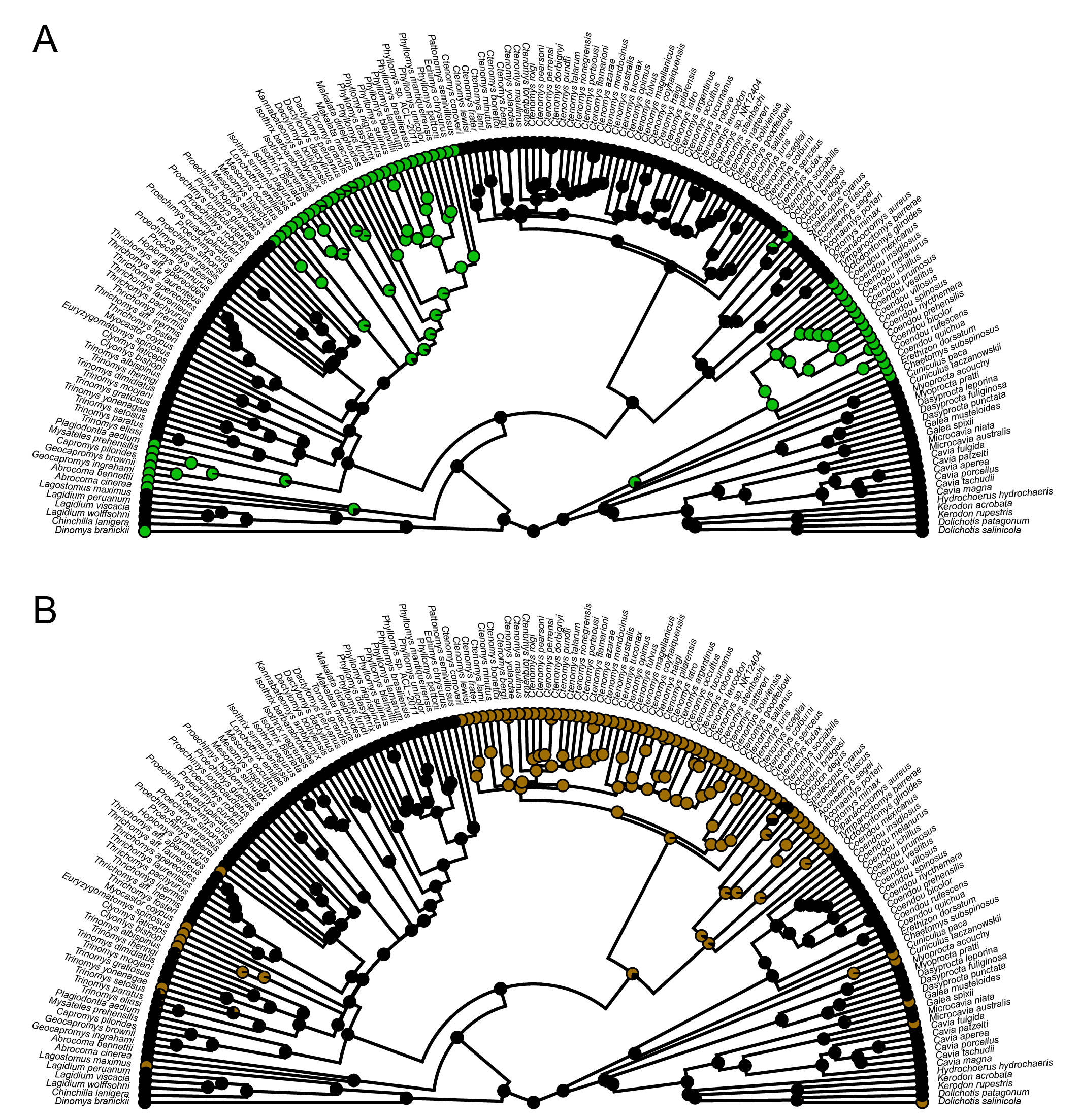
**

**Fig. S1: Raw phylogeny of Caviomorpha from Hedges et al. (2015) with reconstructed lifestyles according to the two regime models.** Pie charts at tips and nodes indicate posterior probability of lifestyles. The pruned phylogeny and mappings for fitting the two Ornstein Uhlenbeck models with two optima is provided in Fig. 4. A) Terrestrial species in black and scansorial species in green. B) Overground species in black and fossorial species in brown.


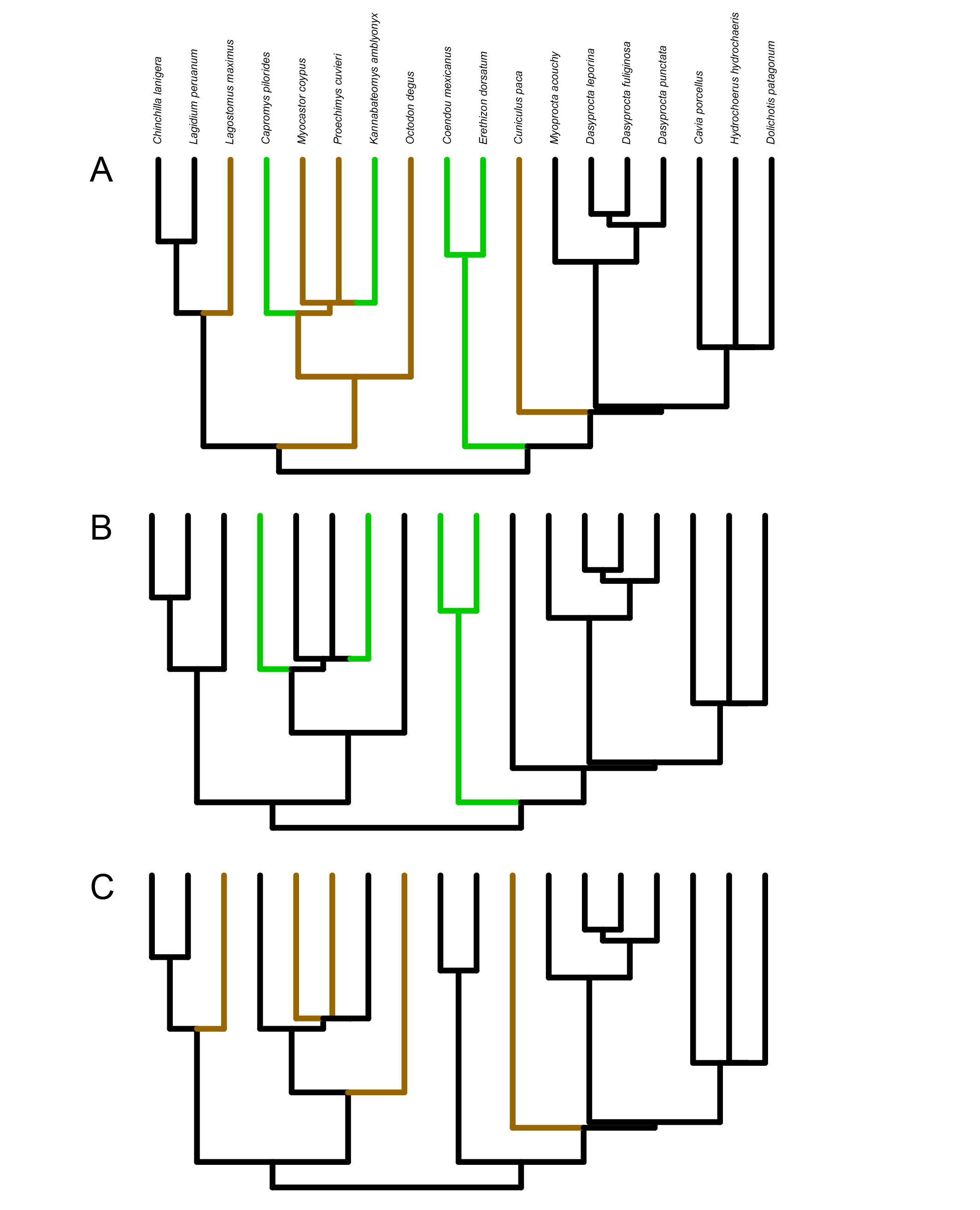


**Fig. S2: Pruned phylogeny with Lifestyles mapped onto the branches according to Fig. 4B-D.** A) OU3 model; cursorial species in black, fossorial species in brown, and scansorial species in green. B) OU2_terr_ model; terrestrial species in black and scansorial species in green. B) OU2_over_ model; overground species in black and fossorial species in brown.


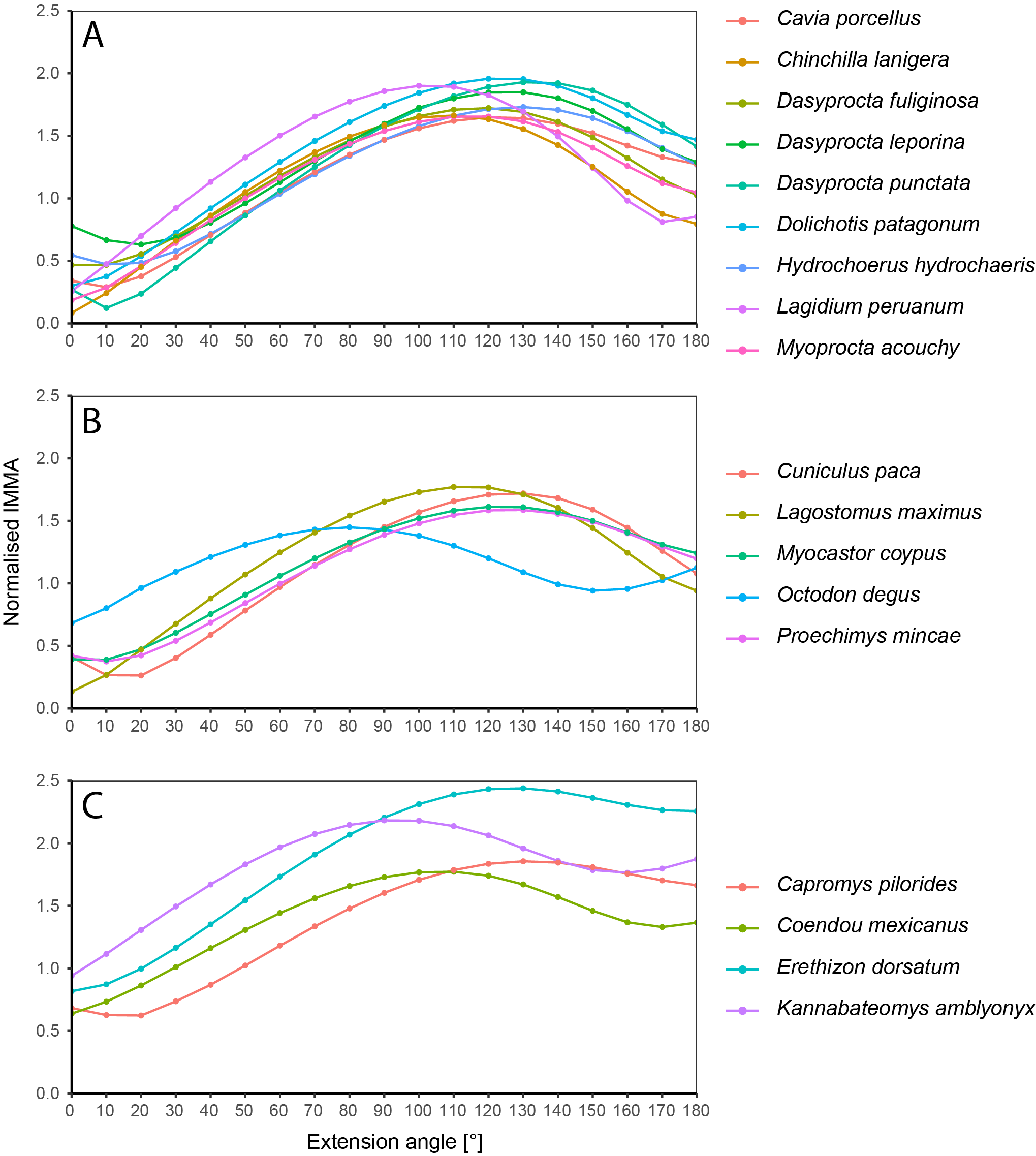
**Fig. S3: Normalised IMMA values for each retraction angle at 30° abduction angle for every modelled species.** Sorted by lifestyle category. A) cursorial, B) fossorial, C) scansorial.
